# The interplay between patients and healthcare professionals in a cross-sectoral setting in connection with the treatment and care of patients with diabetic foot ulcers: a realistic evaluation

**DOI:** 10.1186/s12913-024-11219-1

**Published:** 2024-07-09

**Authors:** Susanne Friis Søndergaard, Jørn Fryd Christensen, Marie Dahl, Milica Drejer, Annette Høgh

**Affiliations:** 1 Vascular Research Unit, Department of Vascular Surgery, Viborg Regional Hospital, Viborg, Denmark; 2 Department of Health, Aarhus University, Aarhus, Denmark; 3 Department of Human and Technology, Roskilde University, RUC, Roskilde, Denmark; 4 VIA University College, Viborg, Denmark; 5 Vascular Research Unit, Department of Surgery, Viborg Regional Hospital, Vibor, Denmark; 6 Department of Clinical Medicine, Aarhus University, Aarhus, Denmark; 7 Cardiac, Thoracic, and Vascular Research Unit, Department of Clinical Research, University of Southern Denmark and Odense University Hospital, Odense, Denmark; 8 Vascular Research Unit and Wound Centre, Department of Surgery, Regional Hospital Viborg Region Central Jutland, Viborg, Denmark; 9 Institute for Clinical Medicine Aarhus University, Aarhus, Denmark

**Keywords:** Patient roles, Realistic evaluation, Cross-sectoral collaboration, Diabetic foot ulcer, Health promotion, Humor

## Abstract

**Background:**

Diabetes-related lower extremity complications such as diabetic foot ulcer (DFU) are a global disability burden. Treatment and care for patients with DFU call for a multisectoral approach that incorporates interdisciplinary care pathways. We aimed to explore the interplay between patients with DFU and healthcare professionals in cross-sectoral settings that address treatment and care and to determine “*what works, for whom, and under what circumstances*”.

**Method:**

The study was designed as a realistic evaluation. The data were generated from September 2022 to March 2023 and drew upon approximately 60 h of participant observation of 14 patients during the treatment and care of DFUs in their homes (primary care) and/or at outpatient clinics (wound specialist clinics in a hospital setting) in a Danish cross-sectoral setting. The Standards for Reporting Qualitative Research (SRQR) were applied in this study.

**Results:**

We identified three illuminating themes that described the interplay between patients with DFU and related healthcare professionals representing both primary and secondary health care systems: (1) humour is a relationship-enhancing element between nurses and patients; (2) support from patients’ coping strategies promotes patient-centeredness and collaboration; and (3) patients and professionals occupy unnegotiated identity roles.

**Conclusion:**

Our study led to a refined programme theory developed through the realistic evaluation process that allows us to propose an answer to the problem of “what works, for whom, and under what circumstances”. The interplay between patients with DFU and healthcare professionals in a cross-sectoral setting for treatment and care is characterised by the use of humour as a relation-enhancing element and by improving support for patient coping strategies, which encourages healthcare professionals to promote health literacy. Future research should examine strategies for negotiating identity roles between patients with DFU and healthcare professionals to enhance collaboration, patient health literacy, and health promotion in cross-sectoral healthcare settings.

## Background

Diabetes mellitus presents a formidable global health challenge, and its associated complications exact a profound toll on individuals and healthcare systems worldwide. Among these complications, diabetic foot ulcers (DFUs) are particularly burdensome and inflict a multitude of adverse physical, psychological, and social effects on individuals with diabetes [1]. As of 2016, DFUs affected an estimated 1.8% of the global populace, highlighting their pervasive nature [2]. Several studies have shown that the ramifications of DFUs extend far beyond mere physical discomfort and cause affected individuals to contend with heightened morbidity and mortality as well as diminished quality of life [3–5].

The lifetime prevalence of DFUs for patients with diabetes ranged from 19 to 34%, with impacted individuals facing significantly curtailed life expectancies compared to their counterparts without DFUs [6]. Moreover, patients with DFUs frequently experience hospitalisation, which exacerbates the strain on healthcare infrastructure. Improving treatment and care in healthcare systems and among patients highlights the importance of efficacious management strategies [5].

Effectively addressing the issues that occur for patients with DFUs requires a collaborative, multidisciplinary approach that enlists the expertise of diverse healthcare professionals to provide treatment and comprehensive care [4–6]. Multidisciplinary teams have emerged as the gold standard for managing and preventing diabetes-related lower-extremity complications; however, previous studies underscore the importance of coordinated interventions across various healthcare domains [7, 8]. Nevertheless, according to a cross-sectional survey from Australia, the successful treatment and management of challenges for patients with DFUs hinge upon close collaboration between patients and healthcare providers with interventions meticulously tailored to individual needs and dispensed across primary care and specialist settings [9].

In agreement with Vo et al. (2021), other studies stress that patients’ health literacy and self-management proficiencies are central to the efficacious management of DFUs. Conversely, inadequate health literacy is a formidable barrier to self-care among individuals with diabetes, indicating the need for interventions geared towards fortifying patients’ empowerment and fostering self-efficacy [10–14]. Encouragingly, health promotion initiatives are promising ways to nurture self-efficacy and effect positive behavioural changes among patients with DFU, emphasising the potential for targeted interventions to improve outcomes [5, 15–19].

Despite the acknowledged importance of collaboration and interaction between caregivers in primary care settings and patients with DFU, qualitative research on this topic is lacking [20, 21]. An understanding of patients’ perceptions of illness and the multiple factors that influence self-care behaviours is imperative for optimising DFU care delivery [22]. However, the extant literature lacks comprehensive insights into these dynamics and underscores the need for further research to bridge this knowledge gap. The involvement and empowerment of patients with DFU can be aligned with healthcare professionals’ agenda, including the promotion of health literacy and health promotion to ensure person-centred care and treatment. In addition, an understanding of the contributory causes of nonadherence to treatment and care among patients with DFU as well as the influence of insufficient health literacy is needed. To our knowledge, a limited number of published studies have addressed the interplay between patients and healthcare professionals with regard to the treatment and care of DFUs in a cross-sectoral setting [20] 2021). This interaction is of utmost importance for ensuring an effective course of wound healing and assisting patients in becoming more aware of their illness and their self-efficacy.

Therefore, the aim of this study was to explore the interplay between patients with DFU and healthcare professionals in a cross-sectoral setting for treatment and care. Furthermore, we aimed to determine *what works, for whom, and under what circumstance*s.

## Methods

This study presents findings from a realistic evaluation (RE) framework, which draws inspiration from the theory-driven approach elucidated by Pawson and Tilley [23]. RE is grounded in the philosophy of critical realism, which perceives the world as an open system comprising intricate structures and layers. Within this framework, practice is understood as a complex interplay of multiple social interactions [24, 25]. Therefore, in an RE study, the objective is to address this complexity and these intricacies by exploring how causation in social practice can be elucidated through the fundamental realist formula for the context-mechanism-outcome configuration (CMOC):

$${\rm{CMOC: mechanism + context = outcome}}$$

CMOC: mechanism + context = outcome.

The analysis relies on an exploration of the interplay between context, mechanism, and outcome, often referred to as CMOC.

**Context** refers to the conditions within which an intervention unfolds. This includes various layers, such as the characteristics of the individuals involved, the local regulations, norms, and traditions within the institutional setting, and the broader social and cultural background. These contextual layers are inherently intricate, interconnected, and constantly evolving [26].

The **mechanisms** elucidate the underlying processes at work and offer insight into the inner workings beneath the surface. The **outcome** includes both the intended and unintended consequences of interventions. It is caused by various mechanisms within different contextual settings [23, 26]. Hence, CMOCs are crafted through an interpretive process that extends beyond mere outcomes. This process not only considers the results but also seeks explanations for why these outcomes were attained. This is achieved by closely examining both the underlying mechanisms and the contextual factors [23, 27].

## Design and method

**Fig. 1:**
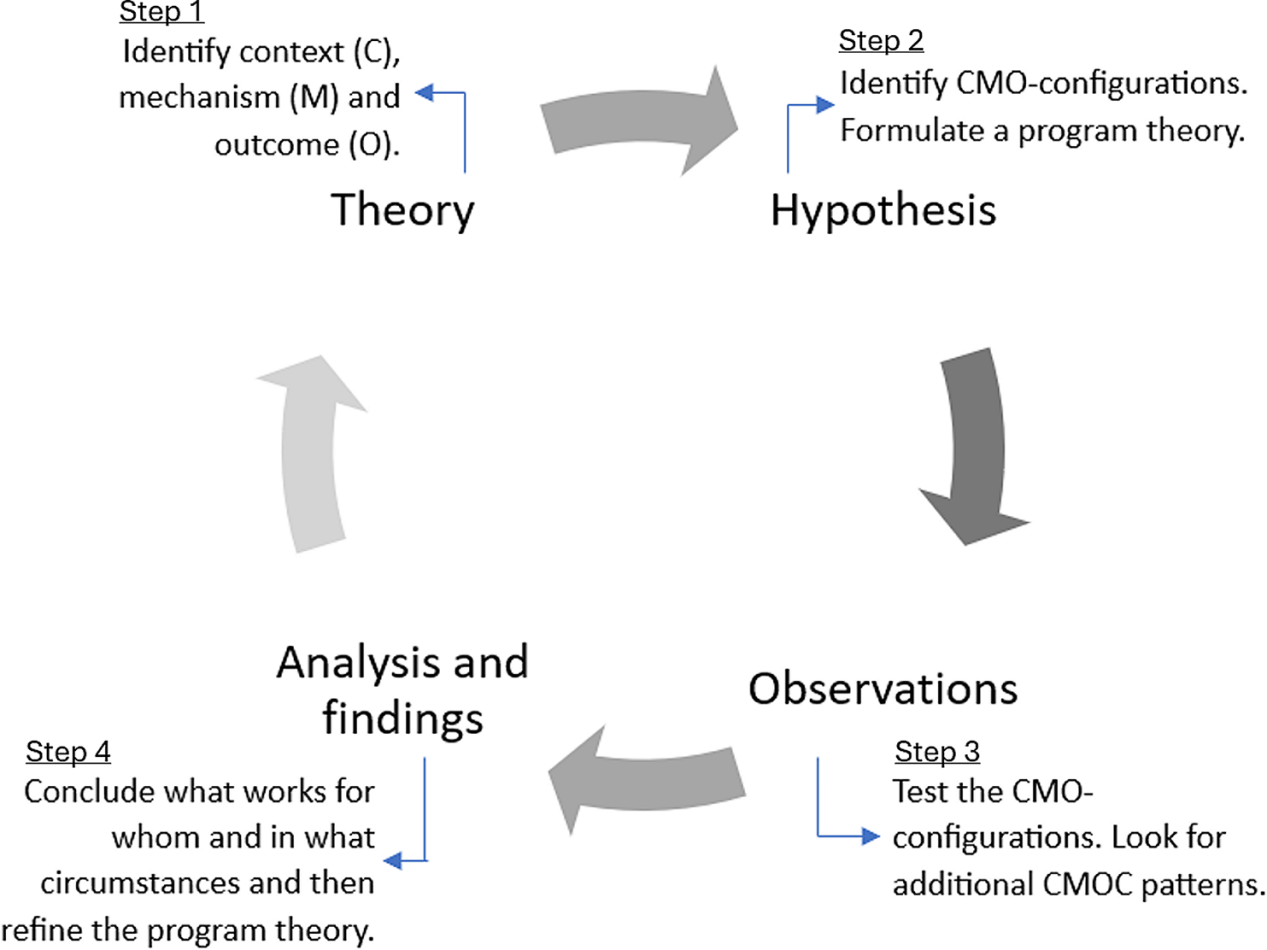
The four-step research process outlined by Pawson and Tilley (1997)

We structured this RE study according to a four-step research process (Fig. 1).

### Theory - step one

**First**, we conducted a state-of-the-art review [28] to elucidate and comprehend social practices within the scope of the evaluation, namely, collaboration between patients and healthcare professionals in a cross-sectoral setting. **Second**, drawing upon the knowledge and insights obtained from the initial step, we formulated a programme theory to steer the study towards identifying and explaining regularities with a focus on discerning contexts, mechanisms, and outcomes. A programme theory in RE can be compared to a hypothesis, encapsulating the previous theory and other knowledge on the topic in a statement. However, in RE, the aim is not only to test the theory but also to improve it.”. [23]p 85.

### Hypothesis/program theory - step two

The programme theory for this study posited *that the interaction between patients with DFU and healthcare professionals within a cross-sector setting of treatment and care is characterised by healthcare professionals’ efforts to promote health literacy*.

### Data generation - step three

**Third**, we collected data during the treatment and care of DFUs in patients’ homes (primary care) and/or at outpatient clinics (wound specialist clinics in a hospital setting) where interactions unfolded between patients and healthcare professionals in cross-sectoral collaboration for DFU care.

Following the recommendation of Hammersley and Atkinson (ethnographic data-generating technique) [29], we used a state-of-the-art literature review and research question to develop a structured observation guide. The content of the observation guide was developed to assist the observer in describing the situation and context of our topics of interest, while also prompting a reflexive approach. It contained questions focusing on what works for whom and under what circumstances. For example, it asked the observer to describe the context of the meeting, what is happening, and what the patient and the healthcare professional are discussing.

Patients were recruited from two Danish wound centres specialising in the treatment and care of individuals with DFU by healthcare professionals trained to identify suitable participants for our study.

We educated healthcare professionals at the centre to identify and allocate all relevant informants according to our inclusion and exclusion criteria. Once identified, the first or last author contacted the patients either in person or by phone to invite them to participate in the study.

Subsequently, the data collectors assessed patient eligibility through a brief interview introducing the study. If patients met our inclusion criteria, they were provided with a concise written overview before being asked to consent to participate.

The inclusion and exclusion criteria for patient participation are detailed in Table 1.

**Table 1:** Inclusion and exclusion criteria for patient participation in the study

**Inclusion criteria**	**Exclusion criteria**
• Adult > 18 years. • With diabetes and diabetic-related foot ulcer(s). • Embedded in an outpatient clinic for treatment and care of the DFU. • Treatment and care for primary health care (nursing and/or general physician).	• Telemedicine treatment and care in general. • Unable to understand and read Danish or English.

In total, 14 patients were enrolled. Data collection occurred during the autumn and winter of 2022/2023 over a period of four months and involved approximately 60 h of participant observation. We observed that treatment and care for patients occurred approximately half the time in the hospital setting and half the time in the primary care setting in the patients’ home or at a healthcare clinic in the primary care setting.

Following the guidelines of participant observation as described by Hammersley and Atkinson, data generation included observing events, listening to conversations, and gathering insights through informal interviews [30]. Due to the initial observations in the study, we acknowledge that formal interviews did not contribute to understanding what works for whom and under what circumstances. Therefore, we decided to refrain from using this data-generating method.

The data were collected by the first author and three nurses who had undergone both theoretical and practical training in ethnographic data-generating techniques, totalling 20 h of instruction. To facilitate systematic data collection and reflection in the field, the nurses responsible for data collection diligently recorded detailed field notes. These notes were transcribed within 24 h of the observation and subsequently discussed with the first author.

### The analysis - step four

In the last step, our objective was to analyse the collected data and to identify connections between the context, mechanism, and outcome within the dataset. To accomplish this, we adhered to the analytical principles delineated by Pawson and Tilley and expanded upon by de Souza. Our overarching aim was to contribute to further generalisations to refine the programme theory [23, 26, 31].

Hence, the analysis comprised three key phases:

1. Identifying outcomes (O) within the data.
2. Determining the context (C) in which these outcomes occurred.
3. Using the insights obtained from the first two steps to discern the underlying mechanisms (M) through interpretation, thereby revealing the factors that precipitated the outcomes within the given context.

**Fig. 2:**
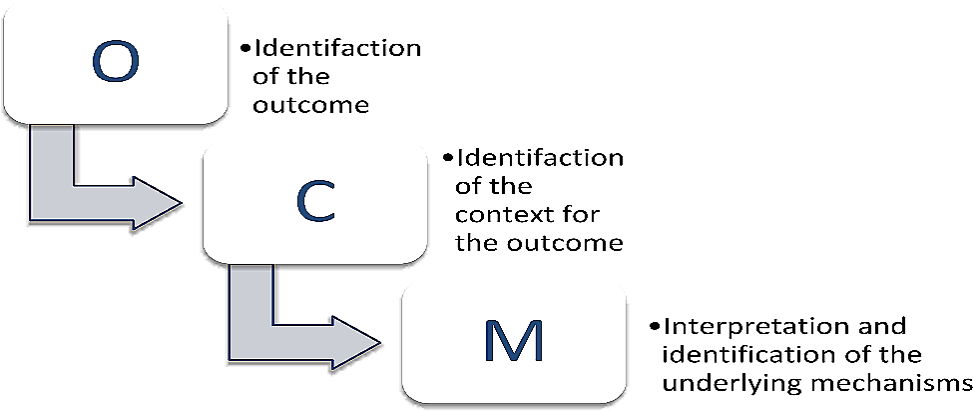
A simplified illustration of the phased analysis process

These analytical principles are rooted in the ontology of critical realism, which is grounded in a stratified view of reality [24]. The analysis was conducted through a distinctive, abstract, and imaginative process. Constructing the CMOC involves the crafting of comprehensive configurations and recognising that various underlying mechanisms can trigger diverse outcomes across different contexts. Moreover, continuous comparison of CMOCs throughout the analysis is essential for identifying interactions among them and establishing connections between shared contexts, outcomes, and mechanisms. The overarching goal of this process was to reveal a nuanced and precise understanding of what interventions are effective for specific groups and under which particular circumstances within the intricate landscape of social practice. Figure 2 shows a breakdown of the phased analysis process.

**Fig. 3:**
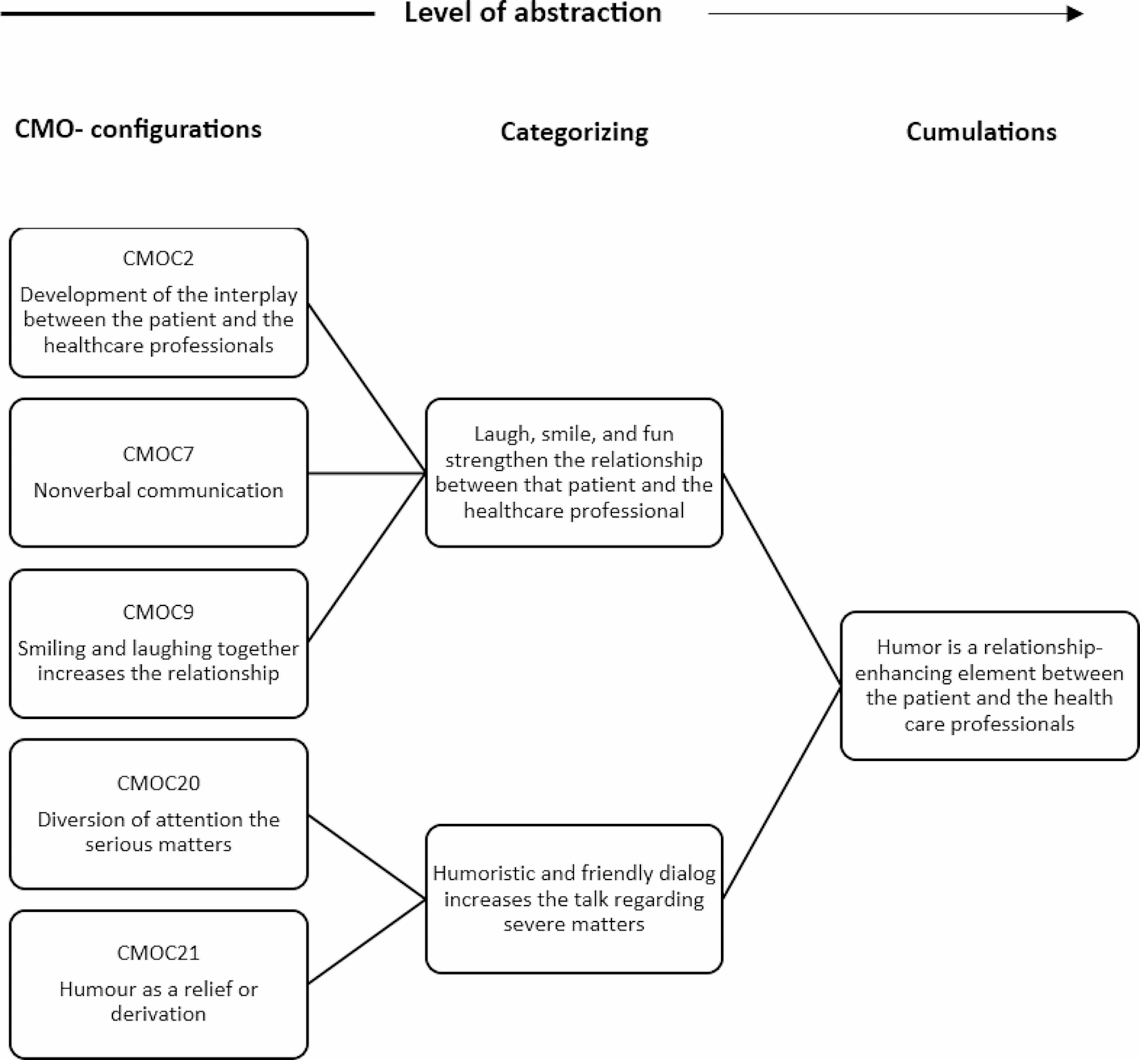
An illustration of the method of gathering and interpreting CMOCs into thematic cumulations

According to the guidelines outlined by Pawson and Tilley, we adhered to the analytical process of cumulation. The analytical and theoretical insights were synthesised and interpreted into thematic, regulative cumulative headings following the principles of analytical induction (see an example in Fig. 3).

In the Results section, we present three thematic cumulations that emerged from our analysis.

These themes reflect the interplay between patients and healthcare professionals in a cross-sectoral setting in which the treatment and nursing care of patients with DFU occurs.

#### Refining the programme theory

Finally, we aimed to determine what works for whom and under what circumstances. The objective of an RE is to develop a refined programme theory and to present the findings of a scientific evaluation [23].

In this study, we present our refined programme theory in the Conclusion section.

### Ethical considerations

This study included records of processing activities for research projects in the Central Denmark Region (record no. 160,406). The study followed the principles of the Declaration of Helsinki and complied with all requirements to ensure the safety of the human volunteers. The heads of the primary care departments and the individual participants provided informed written consent. In adherence with the Danish ethical guideline of the generation of qualitative data, no ethical approval is required if no data refers to identifiable persons or institutions. However, we acknowledge the need for integrity and anonymity of the human volunteers, patients, and professionals in our study. Therefore, the collected observations contained no information that could identify the participants, or the outpatient clinics included in the study. As such no description on the participants is illustrated in this paper.

In addition to reporting the study, we followed the Danish code for integrity in research [32].

Furthermore, our study adopted a qualitative approach and did not involve experiments on humans or the use of human tissue samples/data. As a result, no protocol or ethical approval was deemed necessary.

## Results

From the 52 CMOC, we identified three cumulations that summarised the interplay between patients and healthcare professionals in a cross-sectoral setting for the treatment and care of patients with DFU:

1. Humour is a relationship-enhancing element between patients with DFU and healthcare professionals.
2. The support of coping strategies for patients with DFU promotes person-centeredness and collaboration.
3. Patients with DFU and healthcare professionals responsible for their treatment and care occupy unnegotiated identity roles.

### Humour is a relationship-enhancing element between patients with DFU and healthcare professionals

We found humour to be an underlying mechanism in the interplay between patients and healthcare professionals. An increase in relaxation and openness occurred between patients and healthcare professionals in treatment and care situations that involved the use of a mutual humorous approach. We found that despite severe situations for the treatment and care of patients with DFU, humour created a relaxed atmosphere that led to new and different opportunities for communication between healthcare professionals and patients.

*The patient replies when asked to attend a nursing clinic: “But it’s a long way on a bicycle, especially when you don’t have one and I can’t walk myself”. The nurse agrees, smiling, saying, “You are probably not the most suitable patient to come to a nursing clinic. I will write that you must have wound care at home every fourth day” (RA20.03.B1).*

This quote shows how humorous remarks are “grabbed” and a response is given. However, we also found that a humorous atmosphere can be difficult to clarify conceptually. Some generic characteristic signals in the environment change when the humour becomes mutual. We found that the mechanism for this outcome was characterised by mutual smiling, laughing, and eye contact and that humorous comments were exchanged and especially acknowledged.

*There is talk between the nurse and the patient concerning the importance of using custom-made shoes from the orthotist. The patient smiles when saying, “I think the shoes from the orthotist are very expensive. Maybe I have to be more careful to only use them on selected occasions!” The nurse looks up and says with a laugh in her voice, “You don’t have to be so careful with the money of the municipality. Just use the shoes, and when they are broken I will secure a new pair for you”. The patient says, “Okay” and laughs so much that the chair rocks* (RA06.02CC2).

In addition, we found that the mutual use of humour strengthened the relationship between patients and health care professionals.

*The nurse sits with a foam bandage, looks up at the patient and says with a laugh in her voice, “But it’s not the easiest place to bandage, and then you end up with two wounds…they sit stupidly”. She laughs loudly and liberatingly. The patient and the patient’s wife also burst into laughter, and they laugh for a long time. The patient says, “Can’t you just move them around then?” They laugh again. The nurse continues with the bandage. The patient relaxes in the chair*. After a short while, the nurse looks at the patient, saying, “*Remember it is very important to use the handmade shoe, even though you don’t like to wear it. Otherwise I am afraid that you may get several wounds. That is my fear”. The patient looks her into her eyes and nods* (RA20.03LB).

Humorous dialogue in communication between the patient and the health care professional created a relaxed and safe atmosphere with increased dialogue regarding health promotion for the patient. Conversely, a suspension or break in a relaxed and safe atmosphere was shown in nonverbal signals such as a “serious facial expression” and frowning. When one of the parties deliberately avoids mutual humour, this absence of humour can be used as a guide for communication. For example, when patients try to introduce humour by referring to inappropriate health behaviour, the nurse can send a signal about inappropriateness by failing to reciprocate the humour or by blatantly ignoring statements that are found to be inappropriate.

*The patient says, “I know that I have neuropathy and that I cannot feel that much, but I can feel if I have a stone in my shoe if it is big enough”. He smiles and tries to make eye contact with the nurse, but without success. She doesn’t respond to the patient’s statement (RA20.03LB1).*

This way of ignoring or deliberately not taking part in humorous communication can also be used in reverse.

*The therapist starts taking care of the big toe on the right foot. The patient sits in the chair with his eyes closed and gives a little groan. The therapist says, “I almost cut off the whole nail”. The patient replies, “I’ve never tried that before”. The therapist says, “Then it’s good I have done it before”. She looks up at the patient and smiles. The patient looks back but doesn’t respond (RA09.01LB).*

Our cumulation shows that several different forms of humour can be used with different agendas in the interplay between patients with DFU who are receiving treatment and nursing care and healthcare professionals. Furthermore, humour is used for different purposes in communication between patients and healthcare professionals. However, humour often improves dialogue and patients’ health promotion.

### The support of coping strategies for patients with DFU promotes person-centeredness and collaboration

We found that patients’ need for coping was related to personal and social conditions in relation to the treatment. However, psychological reactions as well as the need for professional knowledge in pragmatic support also influenced patients’ ability to navigate sectoral boundaries in the treatment process.

When the relationship between patients with DFU and healthcare professionals was characterised by collaboration, it enabled specific guidance and knowledge sharing. Moreover, proactively addressing individual patients’ needs for coping support sowed the seeds for collaborative partnerships grounded in mutual understanding.

*“The patient’s wife says, “It’s not so good”, as she looks worried. “We like it when it bleeds,” says the nurse with a loud voice and a small laugh. The physician assures the patient and his wife that it is a “good sign” that there is bleeding from the wound; it means that there is a blood supply and hope for recovery. The patient says, “Well, like that” and “mmm” several times. The wife says they didn’t know it was a good sign: “We have been so worried every time the wound was bleeding at home” (RA23.11MI).*

Sharing professional knowledge and concrete guidance in relation to, for example, changing the dressing, implementing a treatment plan, using medicines, and providing relevant observations at home for patients led to increased competence and person-centred collaboration. The collaboration was supported communicatively by the frequent use of eye contact, nods and smiles during the consultation to accommodate the patient’s needs, and questions and possible misunderstandings could be expressed and explored. In this context, coping supported and guided knowledge sharing. This was realised through a social practice that considered the patient’s understanding, autonomy, and pace in decision-making processes. We found that this had a positive effect on patients’ experience of their influence on clinical decision-making in the cross-sectoral setting.

*The physician points to the foot and illustrates how a toe amputation will be performed. He shows how much of the foot will be removed: “The foot is going to be operated on at this angle, and it is this part that is going to be removed,” he says as he points. He then explains that there is also a risk of poor wound healing after an amputation. The wife adds that it’s more worrying if there are challenges with wound healing afterwards. The physician says they can wait another 3–4 weeks to make the decision about the amputation (*RA22.11MI).

However, if a patient lacked the resources to handle the treatment demands and was unable to navigate the collaboration, a more paternalistic and overbearing approach was used. The underlying mechanism revealed that patients’ complex problems were broken down into smaller, manageable items to achieve a simpler solution. Healthcare professionals assumed responsibility for finding pragmatic solutions by exceeding the usual division of responsibilities in collaboration, such as by initiating interventions, handing out aids, conducting examinations and providing patient information, which normally took place in another setting. Although these kinds of “quick fixes” immediately reduced the complexity of the patient’s situation, we found that this approach could lead to the avoidance of responsibility for supporting the patient’s coping from a long-term perspective.

*The patient does not measure his blood sugar at home as he cannot understand the complicated licensing rules for requiring a blood glucose meter. The nurse hurries to hand him a blood glucose meter, followed by vague instructions on how use it. The patient looks despairing (*RA23.01LB*).*

This liberalistic expectation of being able to take personal responsibility for one’s own health and illness without taking into account the context and patient availability can be the opposite of promoting coping and health literacy.

We found that a cognitive view of knowledge sharing in the collaboration between patients and healthcare professionals, such as by disseminating objective research-based knowledge and information about the consequences of noncompliance with recommendations, could decrease patients’ coping abilities.

*An endocrinologist introduces herself, saying that she wants to talk to the patient about his DM and his possible pancreatic insufficiency, which may coincide with his diarrhoea and DM. The patient says, “Well…!” many times during her report. He does not ask any questions and does not interact with the physician. When she asks him about his long-term blood sugar levels, he says, “What is a long-term blood sugar level?” This question leads to more scientific information from the endocrinologist. The patient looks overwhelmed and does not make eye contact with any of the healthcare professionals in the room (*RA23.01LB*).*

With this form of guidance and knowledge sharing, patients’ coping skills are further challenged by the high degree of complexity of the collaboration.

*The physician: “Are you familiar with § 91 or 94? I can’t remember what it’s called. You can be granted time off to come to the hospital for treatment when you are chronically ill like you”.*

*The patient: “Chronic?”*

*The physician: “Yes, if it can be difficult to get the time to fit in your job”.*

*The patient: “I use holidays and days off”.*

*The nurse:” you need to get hold of your social worker at the municipality”. The patient looks surprised. The nurse: “Which trade union are you in? They can usually help you. The municipality can be a bit difficult to dance with”.*

*The physician: “Yes, they don’t tell it to you because it can cost the municipality money” (*RA23.01LB*).*

We found that this form of support for patients’ coping could give rise to distrust towards cross-sectoral organisations, which could inhibit patients’ experience of coherence. Under these conditions, the result of an overbearing, cognitivist approach to the patient’s coping could be that, rather than coping, the patient resigned.

This cumulation shows that healthcare professionals’ support of patients’ coping in cross-sectoral collaboration can take two forms in relation to coherence: person-centeredness and collaboration in the process. Thus, depending on the patient’s capacity to cope and the healthcare professional’s approach to collaboration, guidance and knowledge sharing can help to increase the patient’s ability to act. However, it can also increase the demands on patients with underdeveloped coping skills. A focus solely on a cognitive, paternalistic, and overbearing approach contributes to quick fixes and carries implicit liberalistic values that can undermine a long-term solution related to the patient’s coping.

### Patients with DFU and healthcare professionals responsible for their treatment and care occupy unnegotiated identity roles.

In collaboration, patients with DFU and healthcare professionals occupy roles that, without any introduction or negotiation, seem to be given in advance.

*The patient hobbles into the out-clinic room and sits down in a chair placed in the middle of the room. The orthotist sits on a stool in front of the patient and begins, without any communication or small talk, to take off the patient’s shoes, stockings, and wound dressing. The patient’s wife sits in a chair with armrests in the corner. It is completely quiet in the room, and no one makes eye contact (RA16.01LB).*

In addition, nonnegotiated roles seem to exist independent of context and setting.

*The door in the hall opens, and a home care nurse enters. There was no knocking at the door, and neither the patient nor his wife greeted her at the door. The home care nurse greets the patient with a hug and a large smile. The nurse acts friendly in her contact with the patient and his wife. She has a relaxed tone and uses jokes in her communication. The nurse shows a clear purpose for her visit. Without any introduction or information, she goes into the bedroom and starts the procedure of preparing the wound care. The patient follows and lies on the bed. The patient’s wife enters the bedroom and sits silently on a chair (HJ16.01LB).*

Moreover, the roles that played out had a clear hierarchy, with healthcare specialists ranking at the top followed by other types of therapists and nurses and patients and families at the lowest level.

An attitude towards the observance of scheduled times for wound treatment and care is another example. For example, patients and their families waited in the outpatient clinic waiting room without any complaints when the agreed-upon time was greatly exceeded. However, if patients disrespected the scheduled time, the nonnegotiated roles triggered the patient to apologise.

The roles were quietly acknowledged by the participants in the collaboration, who accepted their expected roles without hesitation and with a naturalness that supported the distribution of these roles and underpinned the hierarchy.

Language was also used as a facilitator for role distribution and role retention. The analysis showed how special technical terms and scientific language increased the distance to the patient and his or her family.

*An endocrinologist: “Your possible pancreatic insufficiency may coincide with your other symptoms, such as diarrhoea” (*RA23.01LB*).*

In addition, we found that patients assumed and acknowledged their role by not asking critical questions or challenging health professionals.

The patient: “*My occupation is to be a patient – whatever I do, life will go on!*” *(*RA22.11MI).

*The patient says with a low irritable voice, almost to himself: ”I cannot understand why they ask if I am interested in changing the treatment strategy when they just do it anyway” (RA06.01CC2).*

However, we also found that the assigned roles were occasionally challenged. Predefined roles in the hierarchy were challenged if the course suddenly became unexpected.

*The patient: “I am confident about an amputation, but I would like to think about it”.*

*The physician: “We can wait another 3–4 weeks to make the decision”. He looks up, waiting a bit of time before he says, “Okay, this is your decision. You can tell me your decision next time“ (*RA22.11MI).

In this cumulation, we illustrate how the interplay between the patient and the healthcare professional is characterised by unnegotiated predefined roles towards collaboration with regard to the patient’s treatment and care for DFU. Aspects such as placement in the room, the formal hierarchy, and the use of language between the parties maintain the distribution of roles. Furthermore, roles were occasionally challenged, particularly in situations where one of the parties experienced unforeseen development in the collaboration.

## Discussion and conclusion

The aim of this study was to explore the interplay between patients with DFU and healthcare professionals in a cross-sectoral setting for treatment and care. We identified three illuminating cumulations:

First, we found that the shared use of humour promotes relationships and communication between patients and nurses. Humour affects patients’ and nurses’ motivation to communicate about both humorous and serious treatment topics. The importance of humour in nursing has been described several times. A scoping review from 2019 explored the integration of humour in patient‒nurse interactions and identified numerous positive treatment effects from the use of humour in nursing [33]. Sousa et al. indicated that the use of humour facilitates communication by building a therapeutic patient–nurse bond. Humour used in a nursing context promotes, among other things, trust and patient involvement and reduces stress and worry [33].

A similar conclusion was drawn in Astedt-Kurki et al.’s analysis of 17 nurses’ diaries [34]. These authors concluded that the use of humour between nurses and patients enabled both parties to cope with and manage difficult situations. In addition, Astedt-Kurki et al. stressed that humour led to an improvement in the working climate in hospital wards [34].

In our cumulation, we found that the establishment of a loose and relaxed atmosphere is a specific outcome that is observed when humour becomes mutual. Humour triggers mood, and a relaxed atmosphere promotes communication and collaborative relationships between patients and health care professionals. In alignment with our findings, McCraddie et al. (2014) note that a relaxed, bright and cheerful mood can be created through the use of humour to help produce a positive view of a serious treatment process. This relaxed atmosphere is experienced as a much-needed contrast to the gloom and worry that patients may experience in connection with serious illness. The authors note that a positive mood can push the threat of loss aside and increase the space for improvement and a relationship characterised by both serious communication and humorous responses. McCraddie et al. also noted that the use of verbal and nonverbal language that involves not answering, smiling or laughing promotes a dark and silent attitude that supports patients’ perception of the seriousness of the situation, which is consistent with the findings of this study [35]. A lack of response from either the patient or the healthcare professional shows that the humorous dialogue stops in the absence of similar responses. Therefore, the use of humour can be a form of communication that healthcare professionals and patients might avoid if they find it challenging to manage the situation. A thematic synthesis by Jones et al. presented and discussed barriers to the use of humour in nursing. The authors found that even though humour can improve nurse‒patient relationships, it can also have limitations. These authors emphasised that nurses’ individual perceptions present a significant potential barrier that prevents the use of humour in practice. The extent to which nurses use humour is related to personality but is also affected by external and social factors. Hesitancy in its use is influenced by the view that humour is unprofessional [36].

In the second cumulation, our analysis shows that healthcare professionals’ support of patients’ coping in cross-sectoral collaboration can take two forms with regard to coherence and patient-centeredness. Depending on the patient’s capacity to cope and the healthcare professional’s approach to collaboration, guidance and knowledge sharing can increase the patient’s ability to act. The capacity to cope with diabetes has also been discussed in a study by Dahl et al. The authors reported that diabetic patients who feel powerless are often overwhelmed by chronic illness. This can lead to a limited ability to undertake diabetes self-care tasks [22].

A primary multimethod study by Zajdel et al. (2022) noted that coping with diabetes is reflected by two distinct components. The authors showed, in alignment with our findings, that shared appraisal and collaborative coping improve patients’ ability to act and impact diabetes outcomes. However, our findings also illustrate that the patient’s ability to cope in collaboration is associated with distress when diabetes is appraised as an individual problem for the patient. However, higher shared appraisal buffers this effect [21]. Furthermore, patients with weak coping skills were challenged when the focus was solely on a cognitive and overbearing paternalistic approach. These approaches contribute to “quick fixes” and carry implicit liberalistic values that can undermine a long-term solution related to the patient’s coping. This finding is also in line with the study by Zajdel et al. (2018), who showed that communal coping is beneficial to individuals with diabetes. These authors found that providing a positive atmosphere strengthens individuals’ problem-solving skills and, over time, translates into improved well-being and health efficacy [14].

The literature also supports our findings that a person-centred approach improves long-term health outcomes. Studies suggest that increased awareness and attention to individuals with diabetes may help patients become more aware of their illness and increase their self-efficacy. Supportive coping strategies are associated with psychological well-being and quality of life [14, 21, 37].

In the third cumulation, we illustrate how the interplay between the patient and the healthcare professional is characterised by unnegotiated identity roles that define their collaboration. Patients believe they must fulfil an expected role as “*the patient*” to present good management of their own expected duties [37, 38]. However, the role of healthcare professionals in patient-professional relationships is also important. A professional, positive, and nonpaternalistic approach to patients that includes compassion and friendliness can improve feelings of safety, sincerity, and trust in healthcare professionals.

Our study shows that roles can differ between an indulgent paternalistic approach in which the healthcare professional plays the role of being insensitive to the patient and a nonpaternalistic role that is person-centred and friendly in collaboration with the patient. A study by Hirjaba et al. concluded that when health care professionals discourage or accuse patients, this can have a significant negative impact on patients’ morale and lead to decreased commitment to collaboration, self-care, and health literacy [19]. Bester et al. [38] argued that healthcare professionals must be aware of situations in which a patient may be overwhelmed by the severity or the amount of information. Understanding patients’ capacity, experience, and role in healthcare can be useful for improving health literacy. In addition, health literacy may facilitate proactive and health-promoting diabetes self-care [22].

## Strengths and limitations

This study may be limited in scope because it was conducted solely with patients at two centres of excellence in Denmark for patients suffering from DFU. However, these centres receive patients with diabetic foot ulcers from more than 15 municipalities in Denmark.

Furthermore, the scope of cross-sectoral collaboration can be difficult to grasp. We find that RE that includes the contextual perspective is suitable for overcoming this complexity. According to Pawson and Tilley [23] and Pawson and Manzano-Santaella [39], REs can account for the complexity of the social world.

We ensured the transparency and rigor of the RE methodology by meticulously detailing the analytical process and elaborating on how the methodological foundation directed the study. The first and fourth authors collected the data together with trained data collectors, which have improved the richness of the data. In the initial observations of the study, we performed a few individual formal semi-structured interviews. However, in the ongoing analysis of developing CMOSs, we acknowledge that formal interviews did not contribute to understanding “what works for whom and under what circumstances”. Therefore, we decided to refrain from using this data-generating method. However, it is worth noting that this approach recognises the significance of firsthand experiences recalled by participants, whether through direct communication with the observer or interactions with others [24]. Thus, all data were discussed within the research group, in alignment with the RE methodology [23, 31].

## Conclusion

The interplay between patients with DFU and healthcare professionals is characterised by the use of humour as a relationship-enhancing element, the enhancement of patients’ coping strategies and unnegotiated identity roles for both patients and healthcare professionals. This study followed the Standards for Reporting Qualitative Research (SRQR) as described by O’Brien et al. [40].

In summary, this study has led to a refined programme theory developed through the RE process that allows us to propose an answer to the question “*what works, for whom, and under what* circumstances?”:

*In a cross-sectoral setting for treatment and care, the interplay between patients with DFU and healthcare professionals is characterised by the use of humour as a relationship-enhancing element and by improved support for patient coping strategies that promotes healthcare professionals’ agenda to promote health literacy. The unnegotiated identity roles of patients and healthcare professionals challenge collaboration, patient health literacy, and health promotion.*

## Data Availability

The data supporting the findings of this study are accessible through the Surgical Research Unit at the Regional Hospital Viborg. However, due to restrictions imposed as part of a larger research program, the availability of this data is not public. The data written in Danish can be provided upon request to the corresponding author.
